# RETRACTION: Promoting Wound Recovery Through Stable Intestinal Flora: Reducing Post‐Operative Complications in Colorectal Cancer Surgery Patients

**DOI:** 10.1111/iwj.70472

**Published:** 2025-04-02

## Abstract

**RETRACTION:** ChenQ. , WangZ. , and WuB. X. , “Promoting Wound Recovery Through Stable Intestinal Flora: Reducing Post‐Operative Complications in Colorectal Cancer Surgery Patients,” International Wound Journal 21, no. 3 (2024): e14501, 10.1111/iwj.14501.38050345 PMC10898368

The above article, published online on 04 December 2023, in Wiley Online Library (http://onlinelibrary.wiley.com/), has been retracted by agreement between the journal Editor in Chief, Professor Keith Harding; and John Wiley & Sons Ltd. Following an investigation by the publisher, all parties have concluded that this article was accepted solely on the basis of a compromised peer review process. The editors have therefore decided to retract the article. The authors did not respond to our notice regarding the retraction.

**RETRACTION:** Q. Chen , Z. Wang , and B. X. Wu , “Promoting Wound Recovery Through Stable Intestinal Flora: Reducing Post‐Operative Complications in Colorectal Cancer Surgery Patients,” International Wound Journal 21, no. 3 (2024): e14501, 10.1111/iwj.14501.38050345 PMC10898368

The above article, published online on 04 December 2023, in Wiley Online Library (http://onlinelibrary.wiley.com/), has been retracted by agreement between the journal Editor in Chief, Professor Keith Harding; and John Wiley & Sons Ltd. Following an investigation by the publisher, all parties have concluded that this article was accepted solely on the basis of a compromised peer review process. The editors have therefore decided to retract the article. The authors did not respond to our notice regarding the retraction.

